# Anxiolytic and antidepressants’ effect of *Crataegus pinnatifida* (Shan Zha): biochemical mechanisms

**DOI:** 10.1038/s41398-022-01970-6

**Published:** 2022-05-19

**Authors:** Keren Nitzan, Dekel David, Motty Franko, Roni Toledano, Sharon Fidelman, Yaarit Simchon Tenenbaum, Maya Blonder, Shir Armoza-Eilat, Alon Shamir, Moshe Rehavi, Yair Ben-chaim, Ravid Doron

**Affiliations:** 1 Department of Education and Psychology, The Open University, Ra’anana, Israel; 2 Department of Natural and Life Sciences, The Open University, Ra’anana, Israel; 3 Dep. of Physiology and Pharmacology, Sackler Faculty of Medicine, Tel Aviv University, Tel Aviv, Israel; 4 Faculty of Medicine, Technion–Israel Institute of Technology, Haifa, Israel; 5 Mazor Mental Health Center, Akko, Israel; 6 The Dr. Miriam and Sheldon G. Adelson Chair and Center for the Biology of Addictive Diseases, Tel Aviv, Israel

**Keywords:** Depression, Neuroscience

## Abstract

Depression and anxiety disorders are highly prevalent. Selective serotonin reuptake inhibitors (SSRIs) are the current first-line treatment for depression, but they have pronounced limitations. Traditional Chinese medicine can serve as a safe and effective alternative to conventional drugs, particularly since many herbal remedies have already been approved for human use as food additives, making the transition from bench to bedside more efficient. We previously demonstrated that a novel herbal treatment (NHT) induces anxiolytic- and antidepressant-like effects. NHT consists of four herbs: Crataegus pinnatifida (Shan Zha), Triticum aestivum (Fu Xiao Mai), Lilium brownii (Baihe), and the fruit of Ziziphus jujuba (Da Zao). In the current study, we examined the antidepressant-like and anxiolytic-like activities of each individual herb on stressed mice and compared those to the effects of NHT and escitalopram. We show here that Shan Zha is sufficient to produce an anxiolytic and antidepressant-like effect similar to NHT or the escitalopram through activation of 5-HT1A receptor and an elevation in BDNF levels in the hippocampus and Pre-frontal cortex (PFC). Chronic treatment with Shan Zha did not alter serotonin transporter levels in the PFC, as opposed to escitalopram treatment. These results were confirmed in vitro, as none of the herbs blocked SERT activity in Xenopus oocytes. Notably, Shan Zha is sold as a nutritional supplement; thus, its transition to clinical trials can be easier. Once its efficacy and safety are substantiated, Shan Zha may serve as an alternative to conventional antidepressants.

## Introduction

Depression and anxiety disorders are highly prevalent [1–3]. World Health Organization (WHO) estimates that 4.4% of the global population suffers from depressive disorder and 3.6% from an anxiety disorder, making them some of the leading causes of global disability and socioeconomic burden [4–6]. This issue was highlighted during the COVID-19 pandemic, as a recent study suggests that the psychological footprint of COVID‐19 will likely be more substantial than its medical footprint. The development of mood disorders creates a burden that will impede national social and economic recovery even after the pandemic ends [7].

Depression and anxiety are likely to co-occur and share many symptoms and genetic factors [8]. Despite the availability of a wide range of drugs for treating depression and anxiety, most patients fail to achieve complete and sustained remission. Selective serotonin reuptake inhibitors (SSRIs) are the current first-line treatment for depression and anxiety [9–14], but they have pronounced limitations. Notably, they have low success rates, and delayed onset, and are associated with various side effects including sexual dysfunction and weight changes [15–18]. These adverse side effects lead to a high percentage of patients who discontinue treatment. Therefore, there is a great need for a novel antidepressant treatment that has minimal side effects.

In a recent survey, 39% of individuals suffering from depression or anxiety reported they did not receive any conventional treatment and had more supportive attitudes toward natural medications [19], such as herbal remedies. In fact, traditional Chinese medicine has been recognized as an essential part of the drug-development industry ever since Tu Youyou awarded the Nobel prize for developing a malaria treatment based on herbal therapy. Thus, traditional Chinese medicine can serve as a safe and effective alternative to conventional drugs [20–23], particularly since many herbal remedies have already been approved for human use as food additives, making the transition from bench to bedside faster and more efficient.

Previously, we found that treating stressed mice for 3 weeks with a novel herbal treatment (NHT; US Patent No 9,320,772) produced anxiolytic and antidepressant-like effects of a similar magnitude as the SSRI escitalopram [24]. SSRIs reduce the levels of the serotonin transporter (SERT) in different brain regions, such as the hippocampus and prefrontal cortex (PFC) [25, 26], thus extending the effect of 5-HT receptor signaling. However, NHT did not affect SERT levels in the PFC [27]. Its effects were mediated, at least in part, via increased levels of brain-derived neurotrophic factor (BDNF) in the hippocampus and PFC. Importantly, as opposed to escitalopram, NHT did not cause weight gain and sexual dysfunction [24, 27–29]. Furthermore, while escitalopram augmented lipopolysaccharide (LPS)-induced TNFα; NHT abolished LPS-induced interleukin-1β and TNFα peripheral secretion and diminished sickness behavior [30].

NHT consists of four herbs: *Crataegus pinnatifida* (Shan Zha), *Triticum Aestivum* (Fu Xiao Mai), *Lilium brownii* (Baihe), and the fruit of *Ziziphus jujuba* (Da Zao). All these herbs are approved as food additives for human consumption. Shan Zha has some antioxidant and anti-inflammatory capacities [31–34]. Fu Xiao Mai has antioxidant and anti‐inflammatory effects [35] and is rich in flavonoids, amino acids, minerals, and vitamins [36]. Baihe has long been used in Chinese medicine as an anti-inflammatory and antioxidant agent [37, 38]. Lastly, Da Zao induces an anxiolytic-like effect at lower doses and a sedative effect at higher doses in vivo [39, 40]. It is unknown whether the beneficial effect of NHT is mediated by a synergistic effect of all the herbs or if one herb is the source of the anxiolytic and antidepressant-like effects.

Therefore, to identify the active ingredient of NHT and find the bioactive components that exert the anxiolytic effect, we assessed the in vivo behavioral modifications induced by treatment with individual herbs. In addition, it is possible that NHT’s effect on SERT was not observed due to synergistic effects between the herbs, thus we will explore in vitro the possible mechanisms of action of individual herbs, specifically on SERT and the 5-HT_1A_ receptor. Identifying the active ingredient and its mechanism will allow the use of smaller doses and point us toward a more accurate and efficient treatment for depression and anxiety.

## Materials and methods

### Animals

For all in vivo experiments, Male ICR outbred mice (Envigo, Israel) were kept in the vivarium of the Psychobiology Laboratory of the Open University of Israel in Hadassah Ein Kareem medical center. Mice were housed in standard group cages (five mice per cage, each cage contained mice from all experimental groups), kept on a reversed 12 h light/dark cycle (lights on between 1900 and 0700), and given ad libitum access to food and water. All experiments were performed during the dark phase under red light. The care and experimental use of all animals were performed in accordance with the Open University guidelines. All experiments were approved by the Open University’s animal care committee (approval number IL15092, 04/2019).

All in vitro experiments with Xenopus Laevis were performed in accordance with relevant guidelines and regulations and were approved by the Hebrew University’s Animal Care and Use Committee (Ethical approval number NS-11–12909–3).

### Drugs

*Crataegus pinnatifida*, *Triticum aestivu*, *Lilium brownii*, and the fruit of *Ziziphus jujuba* were purchased as freeze-dried granules from KPC Products, Inc (Irvine, CA, USA). Escitalopram was kindly donated by TEVA Ltd (Israel). NHT was prepared by dissolving the four compounds together in saline containing 1% DMSO to give a final concentration of 0.47 mg/ml (each). NHT was administered at a daily dose of 30 mg/kg. Each of its components was administered at a daily dose of 7.5 mg/kg (one-quarter of the complete NHT treatment), and escitalopram was administered at a daily dose of 15 mg/kg (all by i.p. injection) for 3 weeks [24].

### Unpredictable chronic mild stress (UCMS)

This procedure induces anxiety and depression-like behaviors. The procedure was performed during adolescence, starting at the age of 30 days and lasting for 4 weeks, as previously described [24, 27, 41]. Mice were subjected to unpredictable stress using the following stressors: placement in an empty cage with 1 cm of water at the bottom, light/dark cycle inversion, placing the mice in cages with wet sawdust, tilting the cages at 30 degrees, inducing social stress by placing mice in the soiled cages of other mice and restraining the mice. The mice were exposed to each stressor for 4 h (excluding the light/dark cycle inversion, which lasted for 48 h) at different times of the day and at random.

### Study design

One-month-old mice were subjected to 4 weeks of UCMS, after which they were randomly divided into seven treatment groups (12–17 mice per group): Vehicle, Escitalopram, NHT, *Crataegus pinnatifida* (Shan Zha), *Triticum aestivum* (Fu Xiao Mai), Lilium brownii (Baihe), and fruit of *Ziziphus jujuba* (Da Zao). All our behavioral observations and biological examination were conducted in a ‘blind’ fashion, in which the person conducting the test did not know which samples he was working on

**Fig. 1:**
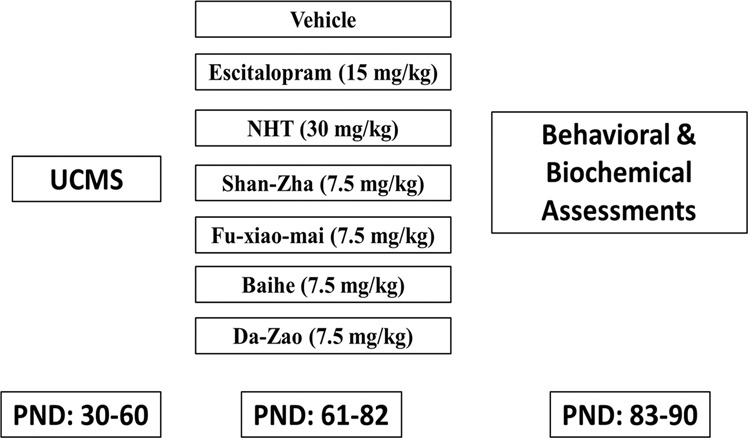
A diagram depicting the study design. At the age of 30 days, mice were subjected to UCMS (4 weeks) and subsequently treated with vehicle, escitalopram, NHT, or the individual NHT components (3 weeks), after which behavioural and biochemical assessment were performed.

All treatment groups received daily treatment for three weeks. Behavioral tests began 24 h after the last treatment. Mice performed 1 test per day and were allowed a 24 h rest between tests. All the tests were done at a monitored room temperature (22–23 °C) under red-light conditions in the dark phase of the animal’s dark/light cycle. Furthermore, mice were allowed to habituate to the room for 30 min before the tests. Twenty-four hours after the final behavioral tests, mice were sacrificed for biochemical assessments (Fig. 1).

### Behavioral assessments

Elevated plus maze (EPM): The EPM test was used to monitor anxiety-like behavior. EPM is based on the natural tendency of mice to avoid open and elevated places and was performed as previously described [42]. The apparatus, situated 40 cm above the floor, consisted of a plus maze with two black plastic closed arms and two opposite open arms. Each mouse was placed in the center of the EPM, and its behavior was video recorded for 5 min. The maze was thoroughly cleaned with ethanol and allowed to dry between subjects to eliminate any odor cues. Anxiety-like behavior was measured by the time the animals spent in the maze’s open, unprotected arm, which is inversely correlated with anxiety.

Tail suspension test (TST): The TST was used to monitor depression-like behavior [27]. Mice were suspended from a horizontal bar by taping the tip of their tail to the bar for 6 min, and the time spent in immobile positions during the last 4 min was recorded. Immobility is correlated with depression-like behavior.

The open-field test: The open-field test was used to monitor motor performance. The apparatus consists of an empty square arena (40 × 40 × 40 cm) that is surrounded by Perspex opaque walls. Each mouse was placed in the center of the arena and video recorded for 5 min. The arena was thoroughly cleaned with ethanol and allowed to dry between subjects to eliminate any odor cues. Locomotor activity was expressed as the percentage of time the mouse was moving in the arena at a velocity above 0.1 pixel/sec.

All the behavioral assays were recorded and analyzed using the Biobserve software.

### Biochemical assessments

Assessment of brain BDNF levels: Tissue samples were obtained as previously described [24]. Shortly, mice were decapitated, and their brains were placed on ice. Serial sections were cut onto slides, and tissue punches of the hippocampus and PFC were taken. Tissue punches were homogenized in cold extraction buffer (Tris-buffered saline, pH 8.0, with 1% NP-40, 10% glycerol, 5 mM sodium metavanadate, 10 mM PMSF, 100 μg/ml aprotinin, and 10 μg/ml leupeptin). Homogenates were acidified with 0.1 M HCl (pH 3.0), incubated at room temperature (22–24 °C) for 15 min, and neutralized (pH 7.6) with 0.1 M NaOH. Homogenates were then microfuged at 7000 × *g* for 10 min. BDNF levels were evaluated using a sandwich enzyme-linked immunosorbent assay as previously described [43].

Assessment of serotonin transporter (SERT) levels: Levels of brain SERT were evaluated using high-affinity [3H]citalopram binding assays. In short, mice were decapitated, and their brains were dissected on ice. Mice brain PFC and hypothalamus were disrupted with Brinkman polytron in 50 vol of buffer (50 mM Tris–HCl, 120 mM NaCl and five mM KCl at a pH of 7.4) and centrifuged (×3) at 30,000 × *g* for 10 min. The pellet was resuspended in the same buffer to yield a final concentration of about 21 mg/ml (wet weight). [3H]Citalopram binding was determined by a standard binding assay that contained 100 μl of brain homogenate, 100 μl [3H]citalopram (0.54 nM), and 300 μl buffer. After a 60 min incubation period at 25 °C, homogenates were diluted in 3 ml of ice-cold buffer and filtered under vacuum through Whatman GF/C glass fiber filters. Filters were washed (×3) with 3 ml of ice-cold buffer, and the radioactivity was measured in scintillation liquid using a β-counter (Packard, Tri-Carb 2100TR). Specific binding was defined as the difference between total [3H]citalopram binding (triplicate samples) and the binding in the presence of 10 μM fluoxetine (duplicate samples). Protein concentration was measured by the method of Lowry et al. [44].

### Functional SERT AND 5-HT_1A_ assays in Xenopus oocytes

#### Preparation of cRNA and oocytes

cDNA plasmids of the two subunits of the G-protein activated inward rectifying K+ channel (GIRK) (GIRK1 and GIRK2), 5-HT_1a_ (kindly provided by Dr. Erhard Wischmeyer from the University of Würzburg, Germany) and SERT (kindly provided by Dr. Walter Sandtner, Medical University of Vienna, Austria) were linearized with the appropriate restriction enzymes and transcribed in vitro using the mMESSAGE mMACHINE Transcription Kit (Invitrogen).

Xenopus laevis oocytes were isolated and incubated in NDE96 solution composed of ND96 (in mM, 96 NaCl, 2 KCl, 1 CaCl2, 1 MgCl2, 5 Hepes, pH adjusted to 7.5 with NaOH), with the addition of 2.5 mM Na+ pyruvate, 100 U/ml penicillin and 100 mg/ml streptomycin [45]. For the 5-HT_1A_ assay, the oocytes were injected with the following cRNAs: 5-HT_1a_ receptor (2 ng/oocyte), GIRK1 and GIRK2 (200 pg/oocyte for each), and Gαi3 (1 ng/oocyte). For the SERT assay, cRNA of SERT (2 ng/oocyte) was injected.

#### Current measurements

The currents were measured 3–4 (5-HT_1a_ assay) or 6–7 (SERT assay) days after cRNA injection and were recorded using a two-electrode voltage-clamp amplifier [46] (Warner OC 725 C amplifier, Warner Instruments, Hamden,CT). The oocyte was placed in the recording bath containing ND96 solution and was impaled with two electrodes pulled from 1.5 mm borosilicate capillaries (Warner instruments). Both electrodes were filled with 3 M KCl solution. The electrode resistances were between 0.5 and 2 MΩ. 5-HT_1A_ receptor-mediated GIRK currents were measured in a 24 mM K + solution (72 mM NaCl, 24 mM KCl, 1 mM CaCl_2_, 1 mM MgCl_2_, 5 mM Hepes, pH adjusted to 7.5 with KOH). pCLAMP10 software (Axon Instruments) was used for data acquisition.

### Statistics

All results are presented as mean ± standard error of the mean. The sample size was determined by power analysis for one-way ANOVA, relying on previous research published by our group [27], conducted in G*Power, using an alpha of 0.05, a power of 0.80, and a large effect size of f = 0.4. All comparisons were carried out by one-way ANOVA and Dunnett post-hoc, after verification that the assumption of the equality of variances between groups was met, except for the SERT levels analysis, which was performed using planned comparison. The level of significance was set at *p* < 0.05.

## Results

### Treatment with Shan Zha reduced depression and anxiety-like behaviors in stressed mice

**Fig. 2:**
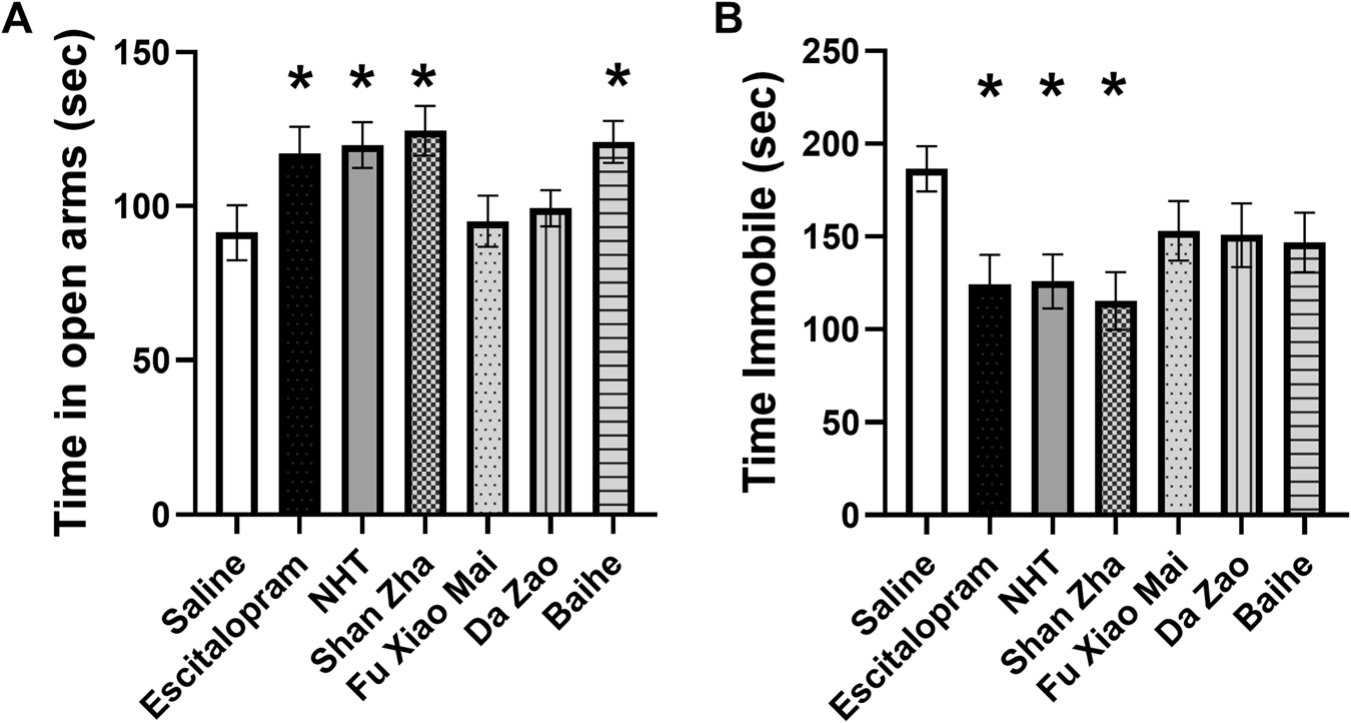
Treatment with Shan Zha reduced depression and anxiety-like behaviors in stressed mice. The effect of treatments on anxiety-like behavior in the EPM. **A** Mice exposed to UCMS and treated with saline spent less time in the open arms of the maze. NHT, escitalopram, Shan Zha, and Baihe treatments reduced anxiety-like behavior. *n* = 13–17 mice per group. *P < 0.05 vs. UCMS + saline group. Effect of treatments on depression-like behavior in the tail suspension test. **B** Mice exposed to UCMS and treated with saline spent more time immobile in the tail suspension test. NHT, escitalopram, and Shan Zha treatments reduced this depression-like behavior. *n* = 12–17 mice per group. *P < 0.05 vs. UCMS + saline group.

Groups of 13–17 mice were exposed to UCMS for one month, treated for three weeks, then assessed for behavior. We used the elevated plus maze (EPM) to test for anxiety-like behaviors. One-way ANOVA revealed an overall significant effect of treatment on anxiety-like behavior [F(6,98) = 3.121, *p* = 0.008]. Dunnett’s post-hoc analysis revealed that Shan Zha– and Baihe-treated mice spent more time in the open arms of the maze in comparison to saline-treated mice (post-hoc *p* = 0.008, *p* = 0.022, respectively). The herbs had a similar magnitude of effect as escitalopram and NHT (p = 0.049 and *p* = 0.023, respectively) (Fig. 2a). Fu Xiao Mai and Da Zao did not have a significant anxiolytic effect. We next tested for depression-like behavior using the tail suspension test. One-way ANOVA revealed an overall significant effect of treatment on depressive-like behavior [F(6,93) = 2.242, *p* = 0.046]. Dunnett post-hoc analysis revealed that Shan Zha–treated mice spent less time immobile compared to saline-treated mice (*p* = 0.005), similar to escitalopram- and NHT-treated mice (*p* = 0.021 and *p* = 0.020, respectively) (Fig. 2b). Fu Xiao Mai, Da Zao, and Baihe did not significantly affect depressive-like behavior.

Because the above assays can be confounded by changes in mouse mobility, we next used the open-field test (OFT) to assess locomotion. No differences in locomotion were observed, suggesting there was no motor impairment and that none of the treatments had sedative or stimulatory effects (data not shown).

### Treatment with Shan Zha increased BDNF levels in the hippocampus and PFC of stressed mice

**Fig. 3:**
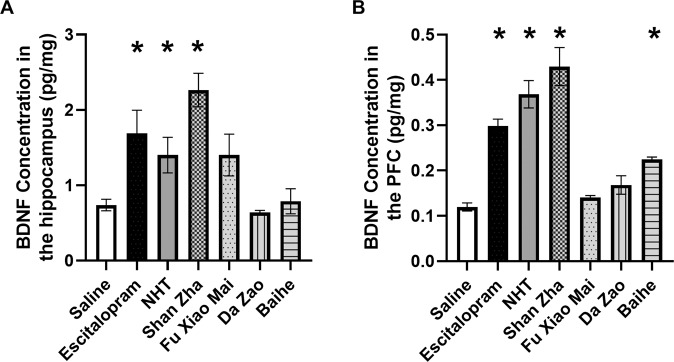
Effect of treatments on BDNF levels. In the hippocampus (**A**), mice treated with NHT, escitalopram, Shan Zha, and Baihe displayed increased BDNF levels compared to saline-treated mice. In the PFC (**B**), mice treated with NHT, escitalopram, and Shan Zha displayed increased BDNF levels compared to saline-treated mice. *n* = 4–8 mice per group. *P < 0.05 vs. UCMS + saline group.

One-way ANOVA revealed an overall significant effect of treatment on hippocampal BDNF levels [F(6,35) = 7.09, *p* = 0.00005]. Shan Zha–treated mice exhibited elevations in hippocampal BDNF levels compared to saline-treated mice (*p* < 0.0001) at a similar magnitude as the escitalopram- and NHT-treated mice (*p* = 0.004, *p* = 0.048, respectively). Fu Xiao Mai, Da Zao, and Baihe did not have a significant effect (Fig. 3A). Similar results were obtained in the PFC (one-way ANOVA: F(6,28) = 26.34, *p* < 0.0001). Shan Zha– and Baihe-treated mice had higher PFC BDNF levels than saline-treated mice (*p* < 0.0001 and *p* = 0.011, respectively), similar to escitalopram- and NHT-treated mice (*p* < 0.0001, *p* < 0.0001, respectively) (Fig. 3B). Fu Xiao Mai and Da Zao did not have a significant effect.

### Treatment with Shan Zha does not affect SERT activity of expression levels

**Fig. 4:**
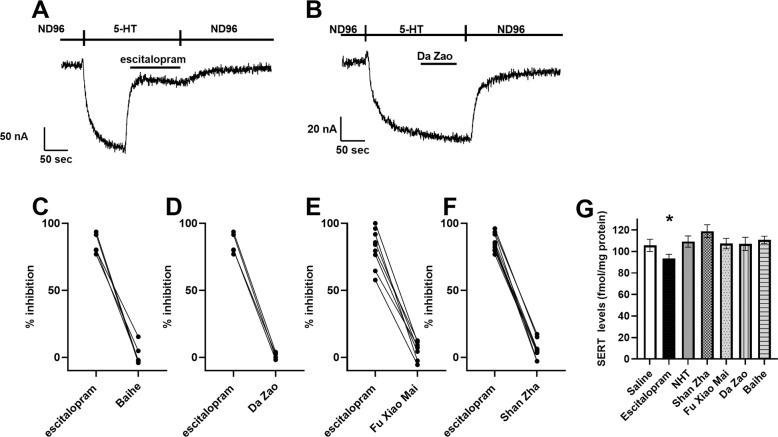
Effect of the different NHT components on SERT activity in Xenopus oocytes. Representative recordings of the effect of escitalopram (**A**) and Da Zao (**B**) on 5-HT induced activation of SERT. Cumulative results from 4–8 oocytes for each component (**C** Baihe, **D** Da Zao, **E** Fu Xiao Mai, **F** Shan Zha). Each two dots connected with a line represent the inhibition of SERT activity by escitalopram (left) and the tested component (right). **G** Effect of the treatments on serotonin transporter (SERT) levels (at 0.54 nM [3H]citalopram) in the PFC. Only the escitalopram-treated mice exhibited reduced SERT levels compared to saline-treated mice. *n* = 4–8 mice per group. *P < 0.05 vs. UCMS + saline group.

We next searched for the mechanism by which Shan Zha exerts its effect. Given the similar magnitudes of effect between Shan Zha and escitalopram on depression-like and anxiety-like behaviors (Fig. 2), we hypothesized that Shan Zha, like escitalopram, affects the levels of SERT. We expressed SERT in a conventional functional expression system, Xenopus oocytes, and assessed the ability of the NHT components to inhibit SERT activity (Fig. 4). The oocyte was voltage-clamped to −60 mV in ND96 solution. Application of 5-HT (5 µM), which SERT transports into the cell, resulted in a steady inward current. To verify that the observed current indeed reflects the transporter’s activation, we treated the oocyte with escitalopram (1 µM), which is a SERT inhibitor. As seen in Fig. 4A, escitalopram blocked the observed current. Next, we assessed the ability of each NHT component to inhibit the activity of SERT, using escitalopram effect as a baseline. Whereas ecitalopram almost completely abolished SERT activity, none of the NHT components had a substantial inhibitory effect on the transporter (Fig. 4B).

We next examined the effect of chronic treatment on SERT in vivo. Planned contrast revealed that SERT levels in the PFC of escitalopram-treated mice were significantly lower than in saline-treated mice (*p* = 0.04). None of the other treatments affected SERT levels (Fig. 4G).

### The effect of Shan Zha on the 5-HT1A receptor

**Fig. 5:**
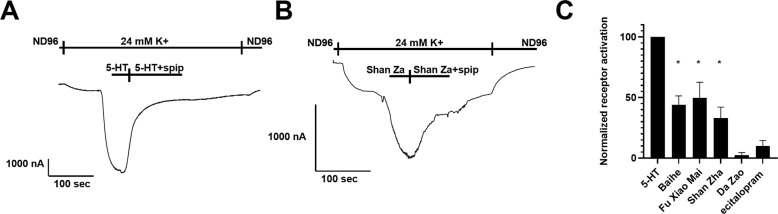
Effect of the NHT components on 5-HT1A activity in Xenopus oocytes. Representative recordings of the effect of 5-HT (**A**) and Shan Zha (**B**) on 5-HT1A-mediated GIRK currents. Spiperone (spip) was used to specifically block the receptor-mediated currents. **C** Cumulative results of the activation of the 5-HT1A receptor by the different components, normalized to the response evoked by 5-HT on the same oocyte. Results are mean ± SEM from 4 to 12 oocytes for each component. Shan Zha, Fu Xiao Mai, and Baihe activated the receptor significantly better than escitalopram (*p* < 0.005).

5-HT1A receptors are widely expressed in the central nervous system and are essential in the pathophysiology of anxiety and depression [47]. 5-HT1A receptor agonist has been shown to produce antidepressant and anxiolytic effects [48]. Therefore, we hypothesized that Shan Zha exerts its phenotypic effect through the 5-HT1A receptor. We used a functional system consisting of the 5-HT1A receptor and GIRK (G-protein activated inward rectifying K+) channel. In this system, the binding of a ligand to the receptor activates its coupled G-protein. The βγ subunits of the G-protein then bind to the GIRK channel and open it. Thus, the current created via this channel measure receptor activation. In each experiment, the oocyte was voltage-clamped to −80 mV in a low K + (2 mM K+) ND96 solution, and the basal GIRK current was created upon replacement of the ND96 by a 24 mM K+ solution. Then, either one of NHT components was applied. To verify that the receptor indeed mediates the evoked current, the 5-HT1A receptor antagonist spiperone (20 µM) was applied and then used as a baseline measurement for a blocked receptor. As a positive control, 5-HT was also applied. We found that three of the four components (Shan Zha, Fu Xiao Mai, and Baihe) induced 5-HT1A-mediated GIRK currents, although less effectively than 5-HT, suggesting that they may be partial 5-HT1A agonists (Fig. 5). Interestingly, in some cases, escitalopram also showed weak 5-HT1A activation.

## Discussion

We previously demonstrated that NHT induces anxiolytic- and antidepressant-like effects by increasing the BDNF levels in the PFC and hippocampus of stressed mice [24, 29, 42, 49]. NHT consists of four herbs: *Crataegus pinnatifida* (Shan Zha), *Triticum aestivum* (Fu Xiao Mai), *Lilium brownii* (Baihe), and the fruit of *Ziziphus jujuba* (Da Zao). In the current study, we examined the antidepressant-like and anxiolytic-like activities of each individual herb and compared those to the effects of NHT and the SSRI escitalopram. We found that Shan Zha treatment reduced depressive-like behavior in the TST, whereas both Shan Zha and Baihe treatments reduced anxiety-like behavior in the EPM. These behavioral effects were similar in magnitude to those observed for NHT and escitalopram. Notably, none of the treatments affected motor functions. Thus, our results indicate that the Shan Zha herb is the most potent component in NHT. This is concordant with our recent study showing that Shan Zha prevents the stress-induced transference of anxiety from dams to pups [50].

SERT is an essential player in the serotonergic system—its uptake terminates 5-HT signaling, and thus it plays a role in determining the duration of 5-HT receptor activation. Escitalopram binding to SERT induces internalization of SERT molecules to intracellular compartments [51], thus allowing 5-HT receptor activation to continue. On the biochemical level, chronic treatment with either Shan Zha or NHT did not alter SERT levels in the PFC, as opposed to escitalopram treatment. These results were confirmed in vitro, as none of the herbs blocked SERT activity in oocytes. This result is in accordance with our previous results regarding NHT and points to a different mechanism by which the Shan Zha or NHT improves depression-like behavior [27]. This could explain the lack of sexual dysfunction seen after NHT treatment as opposed to escitalopram [27], as there is a correlation between impaired sexual behavior after citalopram treatment and elevation of serotonin levels in the PFC [52].

Another important player in mood regulation is the 5-HT_1A_ receptor. It is widely expressed in the central nervous system and is essential in the pathophysiology of anxiety and depression [47]. 5-HT_1A_ receptor agonist produces antidepressant and anxiolytic effects [48]. Partial 5-HT_1A_ agonists, such as Tandospirone and Buspirone, have beneficial effects in the treatment of anxiety and depression [53, 54]. Full agonists, such as 8-OH-DPAT, also exert these beneficial effects; however, they carry the risk of developing Serotonin syndrome [55]. To further test the mechanisms underlying the antidepressant and anxiolytic effects of the four herbs, we examined their interaction with the 5-HT_1A_ receptor in vitro. Our results demonstrate that Baihe, Fu Xiao Mai, and Shan Zha partially activated the receptor. It is important to mention that in our in vivo experiment, none of the treatment groups exhibited serotonergic behaviors, such as backward walking, flat body posture, forepaw treading, head weaving, or tremors.

Our results are in accordance with previous reports. The Shan Zha herb has been shown to have antioxidant and anti-inflammatory capacities in vitro, in vivo, and in clinical trials [31–34]. Shan Zha has many active constituents, with the main phenolics being triterpene acids, hyperoside, isoquercitrin, and chlorogenic acid [56–58]. Hyperoside has an antidepressant effect that may be mediated by HPA (hypothalamus-pituitary-adrenal) axis downregulation [59], reduction of the stress-induced noradrenergic response [60], and upregulation of BDNF in vitro [20]. Isoquercitrin also downregulates the HPA axis function by significantly reducing circulating adrenocorticotropic hormone and corticosterone levels in rats [59], as well as by inhibiting MAO-B activity in vitro [61]. Chlorogenic acid reduces depression-like behavior after corticosterone-induced stress in mice, possibly by inhibiting monoamine oxidase B (MAO-B) and reactive oxygen species (ROS) production [62].

We also saw some beneficial effects of Baihe, although less potent than those of Shan Zha. Baihe has long been used in Chinese medicine as an anti-inflammatory and antioxidant agent, and its compounds mainly include steroidal saponins, sterols, polysaccharides, phenolic glycerides, flavonoids, and alkaloids [37, 38]. In vitro, Baihe has a potent inhibitory effect on MAO-B [63] as well as an interaction with GABA_A_ receptor [64]. In vivo, Baihe has a protective effect in a rat model of chronic mild stress, accompanied by a reduction in noradrenaline levels [65].

BDNF is involved in the pathogenesis of mood disorders [66], and it has been associated with the action of antidepressant and anxiolytic drugs [67]. BDNF is a vital mediator of neuroplasticity, as it modulates multiple processes including axonal and dendritic growth, synaptic plasticity, synaptogenesis, and neurogenesis [43, 68]. The neurotrophic hypothesis of depression posits that downregulation of neurotrophins, predominantly BDNF in the hippocampus and PFC, plays a critical role in the pathogenesis of depression and that upregulation of BDNF is essential for the action of antidepressants [69]. Furthermore, 5-HT_1A_ activation is also associated with BDNF regulation [70, 71]. Notably, we found that BDNF levels in the PFC were elevated by both Shan Zha and Baihe treatments, while hippocampal BDNF levels were elevated only by Shan Zha treatment. These effects were similar to those obtained by NHT and escitalopram treatments. This suggests a role for BDNF in the mechanism underlying the differential behavioral effects of these herbs.

Thus, we show here that the Shan Zha herb (Patent No 275222) is sufficient to produce an anxiolytic and antidepressant-like effect similar to NHT or the SSRI escitalopram through activation of 5-HT_1A_ receptor and an elevation in BDNF levels in the hippocampus and PFC. Few previous studies have demonstrated the biological activities of Shan Zha [32, 59, 72]. Thus, further research is required to reveal the bioactive ingredients within Shan Zha that are responsible for the observed therapeutic effects. Notably, Shan Zha is sold as a nutritional supplement; thus, its transition to clinical trials can be easier. Once its efficacy and safety are substantiated, Shan Zha may serve as an alternative to conventional antidepressants.
